# Congruences for central factorial numbers modulo powers of prime

**DOI:** 10.1186/s40064-016-2028-5

**Published:** 2016-03-31

**Authors:** Haiqing Wang, Guodong Liu

**Affiliations:** Department of Mathematics, Huizhou University, Huizhou, 516007 Guangdong People’s Republic of China

**Keywords:** Central factorial numbers of the first kind, Central factorial numbers of the second kind, Congruence, Stirling numbers, 11B68, 11B73, 05A10, 11B83

## Abstract

Central factorial numbers are more closely related to the Stirling numbers than the other well-known special numbers, and they play a major role in a variety of branches of mathematics. In the present paper we prove some interesting congruences for central factorial numbers.

## Introduction and definitions

Central factorial numbers are more closely related to the Stirling numbers than the other well-known special numbers, such as Bernoulli numbers, Euler numbers, trigonometric functions and their inverses. Properties of these numbers have been studied in different perspectives (see Butzer et al. 1989; Comtet 1974; Liu 2011; Merca 2012; Riordan 1968). Central factorial numbers play a major role in a variety of branches of mathematics (see Butzer et al. 1989; Chang and Ha 2009; Vogt 1989): to finite difference calculus, to approximation theory, to numerical analysis, to interpolation theory, in particular to Voronovskaja and Komleva-type expansions of trigonometric convolution integrals.

The central factorial numbers $t(n,k)$ ($k\in \mathbb Z$) of the first kind and $T(n,k)$ ($k\in \mathbb Z$) of the second kind are given by the following expansion formulas (see Butzer et al. 1989; Liu 2011; Riordan 1968)

1 $$\begin{aligned} x^{[n]}=\sum _{k=0}^n{t(n,k)x^k} \end{aligned}$$

and

2 $$\begin{aligned} x^n=\sum _{k=0}^nT(n,k)x^{[k]}, \end{aligned}$$

respectively, where $x^{[n]}=x(x+\frac{n}{2}-1)(x+\frac{n}{2}-2)\cdots (x+\frac{n}{2}-n+1)$, $n\in \mathbb N_0:= \mathbb N\cup \left\{ 0\right\}$, $\mathbb N$ being the set of positive integers, $\mathbb Z$ being the set of integers.

It follows from (1) that

3 $$\begin{aligned} t(n,k)=t(n-2,k-2)-\frac{1}{4}(n-2)^2t(n-2,k) \end{aligned}$$

with

4 $$\begin{aligned} (x^2-1^2)(x^2-2^2)\cdots (x^2-(n-1)^2)=\sum _{k=1}^nt(2n,2k)x^{2k-2}. \end{aligned}$$

Similarly, (2) gives

5 $$\begin{aligned} T(n,k)=T(n-2,k-2)+\frac{1}{4}k^2T(n-2,k) \end{aligned}$$

with

6 $$\begin{aligned} \frac{x^{2k}}{(1-x^2)\left( 1-(2x)^2\right) \cdots \left( 1-(kx)^2\right) }=\sum _{n=0}^\infty T(2n,2k)x^{2n} \end{aligned}$$

and

7 $$\begin{aligned} k!T(n,k)=\sum _{i=0}^k(-1)^{i}{k\atopwithdelims ()i}\left( \frac{k}{2}-i\right) ^n. \end{aligned}$$

Several papers obtain useful results on congruences of Stirling numbers, Bernoulli numbers and Euler numbers (see Chan and Manna 2010; Lengyel 2009; Sun 2005; Zhao et al. 2014). But only a few of congruences on central factorial numbers for odd prime moduli which can be found in (Riordan 1968, p. 236). For example, let $t_n(x)=\sum _{k=0}^nt(n,k)x^k$, then

8 $$ t_p(x)\equiv x^p-x\pmod {p}, $$

9 $$ t_{p+k}(x) \equiv t_p(x)\cdot t_k(x)\pmod {p}.$$

## Conclusions

In the present paper we prove some other interesting congruences for central factorial numbers. In “[Sec Sec3]” section, some congruence relations for $T(ap^{m-1}(p-1)+r,k)$ modulo powers of prime $p$ are established. For $a$ is odd, $m,k\in \mathbb N$ and $k\le 2^{m-1}a$, we prove that

$$\begin{aligned} k!T(2^{m-1}a,k)\equiv \left\{ \begin{array}{lr} -2^{k-1}\pmod {2^m},\quad k\equiv 0\pmod {4},\\ 2^{k-1}\pmod {2^m},\quad k\equiv 2\pmod {4}. \end{array} \right. \end{aligned}$$

For $$p$$ is odd prime, $$m,a,k\in \mathbb N,r\in \mathbb N_0,k\le p-1$$ and $$ r<p^{m-1}(p-1)$$, in “[Sec Sec3]” section we also show that

$$\begin{aligned} T(ap^{m-1}(p-1)+r,k)\equiv T(r,k)\pmod {p^m}, \quad 1\le r<p^{m-1}(p-1) \end{aligned}$$

and

$$\begin{aligned} k!T(ap^{m-1}(p-1),k)\equiv (-1)^{\frac{k}{2}+1}{k\atopwithdelims ()\frac{k}{2}}\pmod {p^m},\quad \text{ k } \text{ is } \text{ even.} \end{aligned}$$

In “[Sec Sec4]” section, congruences on $t(2ap^m,2k)$ and $T(2n,2ap^m)$ modulo powers of $p$ are derived. Moreover, the following results are obtained: (1) for $a,k,m\in \mathbb N, b\in \mathbb N_0$ and $2^{m-1}a\le k\le 2^{m}a$, we prove a congruence for $t(2^{m+1}a+2b,2k)\pmod {2^m}$; (2) for $a,n,m\in \mathbb N, b\in \mathbb N_0$ and $n\ge 2^{m}a$, we prove a congruence for $T(2n,2^{m+1}a+2b)\pmod {2^m}$; (3) for $p$ is a odd prime number and $a,k,m\in \mathbb N, b\in \mathbb N_0$, we deduce a congruence for $t(2ap^m+2b,2k)\pmod {p^m}$; (4) for $p$ is a odd prime number, $a,n,m\in \mathbb N, b\in \mathbb N_0$, we deduce a congruence for $T(2n,2ap^m+2b)\pmod {p^m}$.

## Congruences for $T(ap^{m-1}(p-1)+r,k)$ modulo powers of prime $p$

### **Theorem 1**

*For *$a$* is odd, *$m,k\in \mathbb N$* and *$k\le 2^{m-1}a$*, we have*

10 $$\begin{aligned} k!T(2^{m-1}a,k)\equiv \left\{ \begin{array}{lr} -2^{k-1}\pmod {2^m},\quad k\equiv 0\pmod {4},\\ 2^{k-1}\pmod {2^m},\quad k\equiv 2\pmod {4}. \end{array} \right. \end{aligned}$$

### *Proof*

Using Euler’s Theorem, $\varphi (2^m)=2^{m-1}$. Therefore, by Fermat’s Little Theorem, we get $c^{\varphi (2^m)}=c^{2^{m-1}}\equiv 1\pmod {2^m}$ if $c$ is odd. Observe that, when $c$ is even, $c^{2^{m-1}}\equiv 0\pmod {2^m}$.

Then by (7), if $k\equiv 0\pmod {4}$, we yield

$$\begin{aligned} k!T(2^{m-1}a,k)= & {} \sum _{i=0}^k(-1)^{i}{k\atopwithdelims ()i}\left( \frac{k}{2}-i\right) ^{2^{m-1}a}\\\equiv & {} \sum _{i=1,~i~\text{ odd }}^k(-1)^{i}{k\atopwithdelims ()i}\\= & {} -2^{k-1}\pmod {2^m}. \end{aligned}$$

If $k\equiv 2\pmod {4}$, we have

$$\begin{aligned} k!T(2^{m-1}a,k) & = \sum _{i=0}^k(-1)^{i}{k\atopwithdelims ()i}\left( \frac{k}{2}-i\right) ^{2^{m-1}a}\\ & \equiv \sum _{i=0,~i~\text{ even }}^k(-1)^{i}{k\atopwithdelims ()i}\\ & = 2^{k-1}\pmod {2^m}. \end{aligned}$$

This completes the proof of Theorem 1.

### *Remark*

By Theorem 1 and (5), we readily get

11 $$\begin{aligned} k!T(2^{m-1}a+2,k)\equiv \left\{ \begin{array}{lr} -k\cdot 2^{k-3}\pmod {2^m},\quad k\equiv 0\pmod {4},\\ k\cdot 2^{k-3}\pmod {2^m},\quad k\equiv 2\pmod {4}. \end{array} \right. \end{aligned}$$

### **Theorem 2**

*For *$$p$$* is odd prime, *$$m,a,k\in \mathbb N,r\in \mathbb N_0,k\le p-1$$* and *$$ r<p^{m-1}(p-1)$$*, we have*

12 $$\begin{aligned} T\left( ap^{m-1}(p-1)+r,k\right) \equiv T(r,k)\pmod {p^m}, \quad 1\le r<p^{m-1}(p-1), \end{aligned}$$

13 $$\begin{aligned} k!T\left( ap^{m-1}(p-1),k\right) \equiv (-1)^{\frac{k}{2}+1}{k\atopwithdelims ()\frac{k}{2}}\pmod {p^m},\quad \text{ k } \text{ is } \text{ even.} \end{aligned}$$

### *Proof*

By Euler’s Theorem and Fermat’s Little Theorem, we get $a^{\varphi (p^m)}=a^{p^{m-1}(p-1)}\equiv 1\pmod {p^m}$ if $(a,p)=1$, where $(a,p)$ is the greatest common factor of $a$ and $p$. Then by (7) and noting that $(k-2i,p)=1$, we get

$$\begin{aligned} k!T\left( ap^{m-1}(p-1)+r,k\right) & = \sum _{i=0}^k(-1)^{i}{k\atopwithdelims ()i}\left( \frac{k}{2}-i\right) ^{ap^{m-1}(p-1)+r}\\ & \equiv \sum _{i=0}^k(-1)^{i}{k\atopwithdelims ()i}\left( \frac{k}{2}-i\right) ^r\\ & = k!T(r,k)\pmod {p^m}. \end{aligned}$$

Observe that $(k!,p)=1$. Hence,

$$\begin{aligned} T\left( ap^{m-1}(p-1)+r,k\right) \equiv T(r,k)\pmod {p^m}. \end{aligned}$$

The proof of (12) is complete. If $r=0$, then $k$ is even. Therefore,

$$\begin{aligned} k!T\left( ap^{m-1}(p-1),k\right) & = \sum _{i=0}^k(-1)^{i}{k\atopwithdelims ()i} \left( \frac{k}{2}-i\right) ^{ap^{m-1}(p-1)}\\ & \equiv \sum _{i=0}^k(-1)^{i}{k\atopwithdelims ()i}-(-1)^{\frac{k}{2}}{k\atopwithdelims ()\frac{k}{2}}\\ & = (-1)^{\frac{k}{2}+1}{k\atopwithdelims ()\frac{k}{2}}\pmod {p^m}. \end{aligned}$$

The proof of (13) is complete. This completes the proof of Theorem 2.

As a direct consequence of Theorem 2, we have the following corollary.

### **Corollary 3**

For $p$ is odd prime, $a,k\in \mathbb N$ and $r\in \mathbb N_0$, we have

14 $$ T(a(p-1)+r,p)\equiv \left\{ \begin{array}{lr} 0\pmod {p},\quad 3\le r\le p-2,\\ 1\pmod {p},\quad r=1. \end{array} \right.$$

15 $$ T(a(p-1)+r,p-1)\equiv \left\{ \begin{array}{lr} 0\pmod {p},\quad 1\le r\le p-1,\\ 1\pmod {p},\quad r=0. \end{array} \right.$$

16 $$ T(p+2,k+2) \equiv T(p,k)\equiv 0\pmod {p},\quad 3\le k\le p-1. $$

17 $$ T(2p+2,k+2)\equiv T(2p,k)\equiv 0\pmod {p},\quad 4\le k\le p-1. $$

### *Proof*

By setting $m=1$ in (12) and using (5), we have

$$\begin{aligned} T(a(p-1)+r,p) & \equiv T(a(p-1)+r-2,p-2)\\  &  \equiv T(r-2,p-2)=0\pmod {p},\quad (3\le r\le p-2 ),\\ T(a(p-1)+1,p)  & \equiv T(a(p-1)-1,p-2)\\  & \equiv T(p-2,p-2)=1\pmod {p}. \end{aligned}$$

The proof of (14) is complete. Setting $m=1$ and $k=p-1$ in (12), we can readily get

$$\begin{aligned} T(a(p-1)+r,p-1)\equiv 0\pmod {p}. \end{aligned}$$

Setting $$m=1$$ and $$k=p-1$$ in (13), and noting that $$(-1)^j{p-1\atopwithdelims ()j}\equiv 1\pmod {p}\quad (j=0,1,2,\ldots ,p-1),$$$$(p-1)!\equiv -1\pmod {p}$$, we have

$$\begin{aligned} T(a(p-1),p-1)\equiv 1\pmod {p}. \end{aligned}$$

The proof of (15) is complete. If $m=1$ and $a=r$ in (12), then

18 $$\begin{aligned} T(rp,k)\equiv T(r,k)\pmod {p}. \end{aligned}$$

Taking $r=1,2$ in (18) and using (5), we immediately get (16) and (17). This completes the proof of Corollary 3.

## Congruences for $t(2ap^m,2k)$ and $T(2n,2ap^m)$ modulo powers of $p$

To establish the main results in this section, we need to introduce the following lemmas.

### **Lemma 4**

*If *$$m\in \mathbb N$$*, then*

19 $$\begin{aligned}&\prod _{i=1}^{2^{m-1}}(1-((2i-1)x)^2)\equiv (1-x^{2})^{2^{m-1}}\pmod {2^m}, \end{aligned}$$

20 $$\begin{aligned}&\prod _{i=1}^{2^{m-1}}(1-(2ix)^2)\equiv 1\pmod {2^m}. \end{aligned}$$

### *Proof*

We prove this lemma by induction on $m$. We see that (19) is true for $m=1$. Assume that it is true for $m=1,2,\ldots ,j-1$. Then

$$\begin{aligned}&\prod _{i=1}^{2^{j}}(1-((2i-1)x)^2)\\&\quad = \prod _{i=1}^{2^{j-1}}(1-((2i-1)x)^2)(1-((2^j+2i-1)x)^2)\\&\quad =\prod _{i=1}^{2^{j-1}}\Big [\left( 1-((2i-1)x)^2\right) ^2-2^{j+1}x^2(2^{j-1}+2i-1)\left( 1-((2i-1)x)^2\right) \Big ]\\&\quad \equiv \left( \prod _{i=1}^{2^{j-1}}(1-((2i-1)x)^2)\right) ^2\pmod {2^{j+1}}. \end{aligned}$$

For any polynomials $A(x)$, $B(x)$, we have $A(x)\equiv B(x)\pmod {2^m}\rightarrow (A(x))^2\equiv (B(x))^2\pmod {2^{m+1}}$, so we obtain the desired result. That is,

$$\begin{aligned} \prod _{i=1}^{2^{j}}(1-((2i-1)x)^2)\equiv \left( \prod _{i=1}^{2^{j-1}}(1-((2i-1)x)^2)\right) ^2\equiv (1-x^{2})^{2^{j}}\pmod {2^{j+1}}. \end{aligned}$$

The proof of (19) is complete. Similarly, we can prove (20) as follows.

$$\begin{aligned} \prod _{i=1}^{2^{j}}\left( 1-(2ix)^2\right) & = \prod _{i=1}^{2^{j-1}}\left( 1-(2ix)^2\right) \left( 1-((2^j+2i)x)^2\right) \\ & = \prod _{i=1}^{2^{j-1}}\left[ \left( 1-(2ix)^2\right) ^2-2^{j+1}x^2(2^{j-1}+2i)\left( 1-(2ix)^2\right) \right] \\ & \equiv \left( \prod _{i=1}^{2^{j-1}}\left( 1-(2ix)^2\right) \right) ^2\pmod {2^{j+1}}\\ & \equiv 1\pmod {2^{j+1}}. \end{aligned}$$

This completes the proof of Lemma 4.

Similarly, we can get the following results.

### **Lemma 5**

*If *$$m\in \mathbb N$$*, then*

21 $$\begin{aligned}&\prod _{i=1}^{2^{m-1}}\left( x^2-(2i-1)^2\right) \equiv (x^{2}-1)^{2^{m-1}}\pmod {2^m}, \end{aligned}$$

22 $$\begin{aligned}&\prod _{i=1}^{2^{m-1}}\left( x^2-(2i)^2\right) \equiv x^{2m}\pmod {2^m}. \end{aligned}$$

We are now ready to state the following theorems.

### **Theorem 6**

*Let *$a,b,k,m\in \mathbb N$* and *$2^{m-1}a\le k\le 2^{m}a$*, then*

23 $$ t(2^{m+1}a,2k) \equiv (-1)^{k-2^{m-1}a}{2^{m-1}a\atopwithdelims ()k-2^{m-1}a}\pmod {2^m}, $$

24 $$ t(2^{m+1}a+2b,2k) \equiv \sum _{j=1}^{k} t(2^{m+1}a,2j)t(2b,2k-2j)\pmod {2^m}. $$

### *Proof*

By (4) and Lemma 5, we find that

$$\begin{aligned} \sum _{k=1}^{2^{m}a}t(2^{m+1}a,2k)x^{2k-2}(x^2-(2^ma)^2)=(x^2-1^2)\cdots (x^2-(2^ma-1)^2)(x^2-(2^ma)^2). \end{aligned}$$

Thus

$$\begin{aligned} \sum _{k=1}^{2^m a}t\left( 2^{m+1}a,2k\right) x^{2k} & \equiv \left( \prod _{i=1}^{2^m}(x^2-i^2)\right) ^a\\ & =  \left( \prod _{i=1}^{2^{m-1}}\left( x^2-(2i-1)^2\right) \prod _{i=1}^{2^{m-1}}\left( x^2-(2i)^2\right) \right) ^a\\ & \equiv \left( x^{2}-1\right) ^{2^{m-1}a}x^{2^{m}a}\\ & = \sum _{k=0}^{2^{m-1}a}(-1)^k{2^{m-1}a\atopwithdelims ()k}x^{2^{m}a+2k}\\ & = \sum _{k=2^{m-1}a}^{2^{m}a}(-1)^{k}{2^{m-1}a\atopwithdelims ()k-2^{m-1}a}x^{2k}\pmod {2^m}. \end{aligned}$$

This completes the proof of (23). For (24), we can prove this as follows.

$$\begin{aligned}&\sum _{k=1}^{2^{m}a+b}t\left( 2^{m+1}a+2b,2k\right) x^{2k-2}\\&\quad =\left( x^2-1^2\right) \cdots \left( x^2-(2^m a)^2\right) \left( x^2-(2^m a+1)^2\right) \cdots \left( x^2-(2^m a+b-1)^2\right) \\&\quad \equiv \left( x^2-1^2\right) \cdots \left( x^2-(2^ma)^2\right) (x^2-1)^2\cdots \left(x^2-(b-1)^2\right)\\&\quad \equiv \sum _{k=1}^{2^m a}t\left( 2^{m+1}a,2k\right) x^{2k}\sum _{k=1}^{b}t(2b,2k)x^{2k-2}\\&\quad =\sum _{k=2}^{2^ma+b}\sum _{j=1}^{k} t\left( 2^{m+1}a,2j\right) t\left( 2b,2k-2j\right) x^{2k-2}\pmod {2^m}. \end{aligned}$$

This completes the proof of Theorem 6.

### *Remark*

Taking $a=1$ and $k=2^{m-1},2^{m-1}+1,2^{m-1}+2$ in (23), we readily get

$$\begin{aligned} t\left( 2^{m+1},2^{m}\right) & \equiv 1\pmod {2^m},\\ t\left( 2^{m+1},2^{m}+2\right) & \equiv 2^{m-1}\pmod {2^m},\\ t\left( 2^{m+1},2^{m}+4\right) & \equiv 3\cdot 2^{m-2}\pmod {2^m},\quad m\ge 3. \end{aligned}$$

### **Theorem 7**

Let $a,b,n,m\in \mathbb N$ and $n\ge 2^{m}a$, then

25 $$\begin{aligned} T\left( 2n,2^{m+1}a\right)\equiv & {} {n-2^{m-1}a-1 \atopwithdelims ()n-2^{m}a}\pmod {2^m}, \end{aligned}$$

26 $$\begin{aligned} T\left( 2n,2^{m+1}a+2b\right)\equiv & {} \sum _{j=0}^{n}T\left( 2j,2^{m+1}a\right) T(2n-2j,2b)\pmod {2^m}. \end{aligned}$$

### *Proof*

By (6) and Lemma 4, we have

$$\begin{aligned} \sum _{n=0}^\infty T\left( 2n,2^{m+1}a\right) x^{2n} & =  \prod _{i=1}^{2^ma}\frac{x^{2}}{(1-(ix)^2)}\\ & \equiv \left( \prod _{i=1}^{2^m}\frac{x^{2}}{(1-(ix)^2)}\right) ^a\\ & \equiv x^{2^{m+1}a}\left( \frac{1}{\prod _{i=1}^{2^{m-1}}(1-((2i-1)x)^2)\prod _{i=1}^{2^{m-1}}(1-(2i)^2)}\right) ^a\\ & \equiv x^{2^{m+1}a}\frac{1}{(1-x^{2})^{2^{m-1}a}}\\ & = \sum _{n=0}^\infty {n+2^{m-1}a-1 \atopwithdelims ()n}x^{2^{m+1}a+2n}\\ & =  \sum _{n=2^ma}^\infty {n-2^{m-1}a-1 \atopwithdelims ()n-2^{m}a}x^{2n}\pmod {2^m}. \end{aligned}$$

This completes the proof of (25). For (26), we can prove this as follows.

$$\begin{aligned} \sum _{n=0}^\infty T\left( 2n,2^{m+1}a+2b\right) x^{2n}= & {} \prod _{i=1}^{2^m a+b}\frac{x^{2}}{\left( 1-(ix)^2\right) }\\\equiv & {} \prod _{i=1}^{2^ma}\frac{x^{2}}{\left( 1-(ix)^2\right) }\prod _{i=1}^{b}\frac{x^{2}}{\left( 1-(ix)^2\right) }\\= & {} \sum _{n=0}^\infty T\left( 2n,2^{m+1}a\right) x^{2n}\sum _{n=0}^\infty T(2n,2b)x^{2n}\\= & {} \sum _{n=0}^\infty \sum _{j=0}^nT\left( 2j,2^{m+1}a\right) T(2n-2j,2b)x^{2n}\pmod {2^m}. \end{aligned}$$

This completes the proof of Theorem 7.

### *Remark*

Taking $a=1$ and $n=2^{m}+1,2^{m}+2$ in (25), we readily get

$$\begin{aligned} T\left( 2^{m+1}+2,2^{m+1}\right) & \equiv 2^{m-1}\pmod {2^m},\\ T\left( 2^{m+1}+4,2^{m+1}\right) & \equiv 2^{m-2}\pmod {2^m},\quad m\ge 3. \end{aligned}$$

### **Lemma 8**

*If *$p$* is a odd prime number and *$m\in \mathbb N$*, then*

27 $$\begin{aligned} \prod _{i=1}^{p^m}(1-(ix)^2)\equiv \left( 1-x^{p-1}\right) ^{2p^{m-1}}\pmod {p^m}. \end{aligned}$$

### *Proof*

Apparently, by Lagrange congruence, we have

$$\begin{aligned} (1-x)(1-2x)\cdots (1-(p-1)x)(1-px)\equiv \left( 1-x^{p-1}\right) \pmod {p} \end{aligned}$$

and

$$\begin{aligned} (1+x)(1+2x)\cdots (1+(p-1)x)(1+px)\equiv (1-x^{p-1})\pmod {p}. \end{aligned}$$

Thus

$$\begin{aligned} (1-x^2)\left( 1-(2x)^2\right) \cdots (1-(px)^2)\equiv \left( 1-x^{p-1}\right) ^2\pmod {p}. \end{aligned}$$

Hence (27) is true for the case $m=1$.

Suppose that (27) is true for some $m\ge 1$. Then for the case $m+1$,

$$\begin{aligned}&\prod _{i=1}^{p^{m+1}}\left( 1-(ix)^2\right) \\&\quad = \prod _{i=1}^{p^{m}}\left( 1-(ix)^2\right) \left( 1-(p^m+ix)^2\right) \left( 1-(2p^m+ix)^2\right) \cdots \left( 1-((p-1)p^m+ix)^2\right) \\&\quad =\prod _{i=1}^{p^{m}}\left[ \left( 1-(ix)^2\right) ^p-\left( 1-(ix)^2\right) ^{p-1}\left( \sum _{j=1}^{p-1}(jp^{m})^2+2jp^mix\right) \right. \\&\qquad \left. + \text{ terms } \text{ involving } \text{ powers } \text{ of } p^{2m} \text{ and } \text{ higher }\right] . \end{aligned}$$

For any prime $p$ and polynomials $A(x)$, $B(x)$, we have $A(x)\equiv B(x)\pmod {p^m}$. This implies that $(A(x))^p\equiv (B(x))^p\pmod {p^{m+1}}$. With $\sum _{j=1}^{p-1}(jp^{m})^2+2jp^mix\equiv 0\pmod {p^{m+1}}$, we obtain the desired result. That is,

$$\begin{aligned} \prod _{i=1}^{p^{m+1}}\left( 1-(ix)^2\right) \equiv \left( \prod _{i=1}^{p^{m}}1-(ix)^2\right) ^p\equiv \left( 1-x^{p-1}\right) ^{2p^{m}}\pmod {p^{m+1}}. \end{aligned}$$

This completes the proof of Lemma 8.

Similarly, we get the following results.

### **Lemma 9**

*If *$p$* is a odd prime number and *$m\in \mathbb N$*, then*

28 $$\begin{aligned} \prod _{i=1}^{p^m}(x^2-i^2)\equiv (x^{p}-x)^{2p^{m-1}}\pmod {p^m}. \end{aligned}$$

### **Theorem 10**

*Let *$p$* is a odd prime number and *$a,b,k,m\in \mathbb N$*, then*

29 $$\begin{aligned}&t(2ap^m,2k)\equiv (-1)^{\frac{2k-2ap^{m-1}}{p-1}}\genfrac(){0.0pt}0{2ap^{m-1}}{\frac{2k-2ap^{m-1}}{p-1}}\pmod {p^m}, \end{aligned}$$

30 $$\begin{aligned}&t(2ap^m+2b,2k)\equiv \sum _{j=1}^{k} t(2ap^m,2j)t(2b,2k-2j)\pmod {p^m}, \end{aligned}$$

*where *$$2k\equiv 2ap^{m-1}\pmod {p-1}$$.

### *Proof*

By (4) and Lemma 9, we find that

$$\begin{aligned} \sum _{k=1}^{ap^m}t(2ap^m,2k)x^{2k-2}(x^2-(ap^m)^2)=(x^2-1^2)\cdots (x^2-(ap^m-1)^2)(x^2-(ap^m)^2). \end{aligned}$$

Thus

$$\begin{aligned} \sum _{k=1}^{ap^m}t(2ap^m,2k)x^{2k} & \equiv \left( \prod _{i=1}^{p^m}(x^2-i^2)\right) ^a\\ & \equiv (x^{p}-x)^{2ap^{m-1}}\\ & = \sum _{k=0}^{2ap^{m-1}}(-1)^k{2ap^{m-1}\atopwithdelims ()k}x^{2ap^{m-1}+(p-1)k}\\ & = \sum _{k=ap^{m-1}}^{ap^{m}}(-1)^{\frac{2k-2ap^{m-1}}{p-1}}{2ap^{m-1}\atopwithdelims ()\frac{2k-2ap^{m-1}}{p-1}}x^{2k}\pmod {p^m}, \end{aligned}$$

where $2k\equiv 2ap^{m-1}\pmod {p-1}$. This completes the proof of (29).

By (4) we get

$$\begin{aligned}&\sum _{k=1}^{ap^m+b}t(2ap^m+2b,2k)x^{2k-2}\\&\quad =(x^2-1^2)\cdots (x^2-(ap^m)^2)(x^2-(ap^m+1)^2)\cdots (x^2-(ap^m+b-1)^2)\\&\quad \equiv (x^2-1^2)\cdots (x^2-(ap^m)^2)(x^2-1)^2)\cdots (x^2-(b-1)^2)\\&\quad =\sum _{k=1}^{ap^m}t(2ap^m,2k)x^{2k}\sum _{k=1}^{b}t(2b,2k)x^{2k-2}\\&\quad =\sum _{k=2}^{ap^m+b}\sum _{j=1}^{k} t(2ap^m,2j)t(2b,2k-2j)x^{2k-2}\pmod {p^m}. \end{aligned}$$

This completes the proof of Theorem 10.

### *Remark*

Taking $a=1$ and $2k=2p^{m-1},2p^{m-1}+(p-1),2p^{m-1}+2(p-1)$ in (29), we readily get

31 $$\begin{aligned}&t(2p^{m},2p^{m-1})\equiv 1\pmod {p^m}, \end{aligned}$$

32 $$\begin{aligned}&t(2p^{m},2p^{m-1}+(p-1))\equiv -2p^{m-1}\pmod {p^m}, \end{aligned}$$

33 $$\begin{aligned}&t(2p^{m},2p^{m-1}+2(p-1))\equiv 2p^{2m-2}-p^{m-1}\pmod {p^m}. \end{aligned}$$

Obviously,

34 $$\begin{aligned}&t(2p,2)\equiv 1\pmod {p}, \end{aligned}$$

35 $$\begin{aligned}&t(2p,p+1)\equiv -2\pmod {p}. \end{aligned}$$

If $u,v\in \mathbb N,1\le u<2p^v$. By setting $m=1,a=p^v,2k=2p^v+u(p-1)$ in (29), and noting that ${p\atopwithdelims ()j}\equiv 1\pmod {p}\quad (j=0,1,2,\ldots ,p-1)$ with ${ip+r\atopwithdelims ()jp+s}\equiv {i\atopwithdelims ()j}{r\atopwithdelims ()s}\pmod {p}\quad (i\ge j)$, we have

36 $$\begin{aligned} t(2p^{v+1},2p^v+u(p-1))\equiv (-1)^u{2p^v\atopwithdelims ()u}\equiv 0\pmod {p}. \end{aligned}$$

The following corollary is a direct consequence of Theorem 10.

### **Corollary 11**

Let $p$ be a odd prime and $\alpha$ be a positive integer. Then for any $1\le k\le p^{\alpha }(p-1)$, we have

37 $$\begin{aligned} t(p^{\alpha }(p-1),2k)\equiv \left\{ \begin{array}{lr} 1\pmod {p},\quad 2k\equiv 0\pmod {p^{\alpha -1}(p-1)},\\ 0\pmod {p},\quad \text{ otherwise.} \end{array} \right. \end{aligned}$$

### *Proof*

Let $m=1, 2a=p^{\alpha -1}(p-1)$ in (29) of Theorem 10. Then we have

$$\begin{aligned} \sum _{k=1}^{\frac{p^{\alpha }(p-1)}{2}}t(p^{\alpha }(p-1),2k)x^{2k} \equiv \sum _{j=0}^{p^{\alpha -1}(p-1)}(-1)^j{p^{\alpha -1}(p-1)\atopwithdelims ()j}x^{p^{\alpha -1}(p-1)+(p-1)j}\pmod {p}. \end{aligned}$$

By the Lucas congruence, we obtain

$$\begin{aligned} {p^{\alpha -1}(p-1)\atopwithdelims ()j}\equiv \left\{ \begin{array}{lr} \genfrac(){0.0pt}0{p-1}{\frac{j}{ p^{\alpha -1}}}\pmod {p},\quad j\equiv 0\pmod {p^{\alpha -1}},\\ 0\pmod {p},\quad \text{ otherwise.} \end{array} \right. \end{aligned}$$

With

$$\begin{aligned} (-1)^j{p-1\atopwithdelims ()j}\equiv 1\pmod {p}\quad (j=0,1,2,\ldots ,p-1), \end{aligned}$$

we can deduce

$$\begin{aligned} \sum _{k=1}^{\frac{p^{\alpha }(p-1)}{2}}t(p^{\alpha }(p-1),2k)x^{2k} & \equiv \sum _{j=0}^{p-1}(-1)^j{p^{\alpha -1}(p-1)\atopwithdelims ()p^{\alpha -1}j}x^{p^{\alpha -1}(p-1)(j+1)}\\ & \equiv \sum _{j=1}^{p}(-1)^{j-1}{p-1\atopwithdelims ()j-1}x^{p^{\alpha -1}(p-1)j}\\ & \equiv \sum _{j=1}^{p}x^{p^{\alpha -1}(p-1)j}\pmod {p}, \end{aligned}$$

which is obviously equivalent to (37).

The following theorem includes the congruence relations for $T(2n,2ap^m)$ and $T(2n,2ap^m+2b$.

### **Theorem 12**

*If *$p$* is a odd prime number, *$a,b,n,m\in \mathbb N$*, then*

38 $$\begin{aligned} T(2n,2ap^m)\equiv {\frac{2n-2ap^{m-1}}{p-1}-1 \atopwithdelims ()\frac{2n-2ap^{m}}{p-1}}\pmod {p^m} \end{aligned}$$

*and*

39 $$\begin{aligned} T(2n,2ap^m+2b)\equiv \sum _{j=0}^{n}T(2j,2ap^m)T(2n-2j,2b)\pmod {p^m}, \end{aligned}$$

*where *$$2n\equiv 2ap^m\pmod {p-1}$$.

### *Proof*

By (6) and Lemma 8, we have

$$\begin{aligned} \sum _{n=0}^\infty T(2n,2ap^m)x^{2n} & = \prod _{i=1}^{ap^m}\frac{x^{2}}{(1-(ix)^2)}\\ & \equiv \left( \prod _{i=1}^{p^m}\frac{x^{2}}{(1-(ix)^2)}\right) ^a\\ & \equiv x^{2ap^m}\frac{1}{(1-x^{p-1})^{2ap^{m-1}}}\\ & = \sum _{n=0}^\infty {n+2ap^{m-1}-1 \atopwithdelims ()n}x^{2ap^{m}+(p-1)n}\\ & = \sum _{n=ap^m}^\infty {\frac{2n-2ap^{m-1}}{p-1}-1 \atopwithdelims ()\frac{2n-2ap^{m}}{p-1}}x^{2n}\pmod {p^m}. \end{aligned}$$

This completes the proof of (38). For (39), we can prove this as follows.

$$\begin{aligned} \sum _{n=0}^\infty T(2n,2ap^m+2b)x^{2n}= & {} \prod _{i=1}^{ap^m+b}\frac{x^{2}}{(1-(ix)^2)}\\\equiv & {} \prod _{i=1}^{ap^m}\frac{x^{2}}{(1-(ix)^2)}\prod _{i=1}^{b}\frac{x^{2}}{(1-(ix)^2)}\\= & {} \sum _{n=0}^\infty T(2n,2ap^m)x^{2n}\sum _{n=0}^\infty T(2n,2b)x^{2n}\\= & {} \sum _{n=0}^\infty \sum _{j=0}^nT(2j,2ap^m)T(2n-2j,2m)x^{2n}\pmod {p^m}. \end{aligned}$$

This completes the proof of Theorem 12.

### *Remark*

Taking $a=1$ and $2n=2p^{m}+(p-1),2p^{m}+2(p-1)$ in (38), we have

40 $$\begin{aligned}&T(2p^{m}+(p-1),2p^{m})\equiv 2p^{m-1}\pmod {p^m}, \end{aligned}$$

41 $$\begin{aligned}&T(2p^{m}+2(p-1),2p^{m})\equiv 2p^{2m-2}+p^{m-1}\pmod {p^m}. \end{aligned}$$

Obviously,

42 $$\begin{aligned}&T(3p-1,2p)\equiv 2\pmod {p}, \end{aligned}$$

43 $$\begin{aligned}&T(4p-2,2p)\equiv 3\pmod {p}. \end{aligned}$$

If $u\in \mathbb N_0,v\in \mathbb N$. By setting $m=1,a=p^{v-1},2n=2p^{u+v}$ in (38), and noting that ${ip+r\atopwithdelims ()jp+s}\equiv {i\atopwithdelims ()j}{r\atopwithdelims ()s}\pmod {p}\quad (i\ge j)$, we have

$$\begin{aligned} T(2p^{u+v},2p^v) & \equiv {2p^{v-1}\sum _{i=0}^{u} p^i-1\atopwithdelims ()2p^{v-1}-1} \\ & = \frac{1}{\sum _{i=0}^{u} p^i}{2p^{v-1}\sum _{i=0}^{u} p^i\atopwithdelims ()2p^{v-1}}\\  & \equiv \frac{1}{\sum _{i=0}^{u} p^i}{2\sum _{i=0}^{u} p^i\atopwithdelims ()2} \\ & \equiv 1\pmod {p}. \end{aligned}$$

That is,

44 $$\begin{aligned} T(2p^{u+v},2p^v)\equiv 1\pmod {p}. \end{aligned}$$
